# One-factor sex determination evolves without linkage between feminizing and masculinizing mutations

**DOI:** 10.1098/rspb.2024.0693

**Published:** 2024-07-10

**Authors:** Michael F. Scott, Simone Immler

**Affiliations:** ^1^ School of Biological Sciences, University of East Anglia, Norwich Research Park, Norwich NR4 7TJ, UK

**Keywords:** sex chromosome, evolution, dioecy, hermaphrodite, model

## Abstract

The evolution of separate sexes from cosexuality requires at least two mutations: a feminizing allele to cause female development and a masculinizing allele to cause male development. Classically, the double mutant is assumed to be sterile, which leads to two-factor sex determination where male and female sex chromosomes differ at two loci. However, several species appear to have one-factor sex determination where sexual development depends on variation at a single locus. We show that one-factor sex determination evolves when the double mutant develops as a male or a female. The feminizing allele fixes when the double mutant is male, and the masculinizing allele fixes when the double mutant is female. The other locus then gives XY or ZW sex determination based on dominance: for example, a dominant masculinizer becomes a Y chromosome. Although the resulting sex determination system differs, the conditions required for feminizers and masculinizers to spread are the same as in classical models, with the important difference that the two alleles do not need to be linked. Thus, we reveal alternative pathways for the evolution of sex determination and discuss how they can be distinguished using new data on the genetics of sex determination.

## 1. Introduction

Male and female sexual functions can be carried out by separate individuals (hereafter referred to as dioecy) or the same individual (hereafter cosexuality). Almost all animals and flowering plants are either dioecious or cosexual, and transitions between the two systems evolved on numerous occasions [1–3]. Dioecious species with genetic sex determination generally either have XY or ZW sex chromosomes, depending on whether males or females are heterozygous at the sex-determining locus [4,5]. Here, we focus on the evolution of dioecy and genetic sex determination from ancestral cosexuality and thus develop an alternative scenario for the evolution of nascent sex chromosomes.

At least two mutations are required for the transition from cosexuality to dioecy: one that causes female development and one that causes male development [6–8]. These two mutations have classically been called ‘male-sterility’ and ‘female-sterility’ mutations, respectively [7–11], where the phenotype of the double mutant is assumed to be completely sterile. To prevent the production of sterile double mutants, the male- and female-sterility mutations must appear in linkage with one another [9], perhaps even in the same gene [12,13], or linkage must evolve through recombination suppression [7,14,15]. Furthermore, the two mutations must have opposite dominance for dioecy to evolve (e.g. a recessive male-sterility mutation and a dominant female-sterility mutation); otherwise, cosexual or sterile types are produced. The resulting system of linked male- and female-sterility alleles at two loci with opposite dominance is known as two-factor sex determination because genetic variation must be maintained at two loci [16].

The genomic architecture of sex determination systems has been studied in a range of plants [16–18] and animals [4,10]. While two-factor sex determination appears to exist in several plant species, recent results have shown that male/female development in other species depends on genetic variation at a single locus, which is called one-factor sex determination [16,19–21]. Most animals with genetically determined dioecy also have one-factor sex determination [4,22]. Our study is motivated by the observation that some species that have recently evolved dioecy from cosexuality have one-factor sex determination, in disagreement with the model of linked male- and female-sterility alleles on new sex chromosomes.

In revisiting classic models, we retain their assumptions about the evolutionary forces that drive transitions from cosexuality to dioecy, the most prominent of which are resource allocation and selfing [6,23]. In resource allocation theory, gain curves describe the relationship between the resources invested in male/female function and male/female fertility [24]. These curves can include a minimum investment [25,26], temporal separation of investment [27,28] or selfing [29]. In the case of large effect mutations that convert cosexual individuals into males or females, gain curves translate into a single parameter that describes the corresponding increase in male/female fertility, which is called ‘compensation’ for the loss of one sexual function [9,11,30]. Selfing (mating between the male and female parts of the same cosexual individual) is a particularly important factor because it heavily influences the amount of compensation required for dioecy to evolve. It is often assumed that the amount of male sperm/pollen used for self-fertilization is negligible. Thus, cosexuals transmit both maternal and paternal alleles to their offspring through selfing while retaining the ability to sire offspring by outcrossing, creating a transmission advantage [31]. The same phenomenon means that males are at a disadvantage among selfing cosexuals because they must compete to fertilize a relatively small number of outcrossed offspring produced by cosexuals. Thus, dioecy is primarily thought to have evolved via the evolution of females first and then males [9,32]. A cost of selfing is increased homozygosity, where offspring can be homozygous for recessive deleterious mutations and hence suffer from inbreeding depression [33]. Unisexual males or females cannot self-fertilize and therefore their offspring do not suffer from inbreeding depression. Thus, selfing and inbreeding depression among cosexuals can help dioecy to evolve because less compensation is required for males/females to have an advantage over the ancestral cosexuals [9,11,34].

The crucial feature of our model is that we allow the double mutant to develop as either a female or a male, rather than assuming it is sterile. This can happen because the masculinizing and feminizing alleles affect genes in the same biochemical pathway or regulatory network [35]. In animals, master sex determination genes generally have essential functions in the same sexual development pathway [36,37]. In flowering plants, several opportunities for masculinizing and feminizing alleles to interact have been highlighted. In the Caucasian persimmon, *Diospyros lotus* [38,39], and in *Populus* species [40], alleles coding for small RNAs determine sex by suppressing the action of genes required for female development, whereas in spinach, *Spinacia oleracea*, the feminizing pathway represses the masculinizing pathway [41]. Ancestral monoecy, where a cosexual individual bears separate male and female flowers, may also allow double mutants to be fertile [16,21]. For example, a feminizer could express a hormonal signal that triggers female flower development across the whole plant, and a masculinizing mutation might cause insensitivity to that hormone, which makes the double mutant male [16,21]. Regardless of the molecular details, the principle is that masculinizing and feminizing double mutants can develop as males or females because they interact in some way.

Here, we show that one-factor sex determination evolves on new sex chromosomes when the double mutant develops as a male or female instead of being sterile (which is a special case of our model). We consider mutations that are fully masculinizing/feminizing and also fully dominant, while previous work has considered smaller effect alleles [11] or evolving dominance [42] without focusing on the fertility of double mutants. We find that one locus will remain polymorphic and the masculinizing/feminizing allele at the other locus will go to fixation. That is, two mutations spread, but only one remains polymorphic. The evolution of XY or ZW sex determination depends on whether a dominant or recessive masculinizer or feminizer still segregates. We find that the conditions (in terms of selfing rate, inbreeding depression and compensation) for the masculinizer and feminizer to spread are the same as in the classic sterility model, with the difference that the two loci do not need to be linked.

## 2. Model

We track the diploid genotypes at two loci, each with two alleles, *F* and *f* and *M* and *m*. The genotype *ffmm* corresponds to a cosexual individual, and *F* and *M* are mutant feminizing and masculinizing alleles that instead cause the individual to develop as a female or a male, respectively. We note that *F* and *M* are alleles causing female and male development, respectively, rather than female- and male-sterility alleles, which would instead make carriers male and female, respectively, as in the notation of Bachtrog *et al*. [10]. Furthermore, capitalization does not indicate dominance in our notation because we allow masculinizing and feminizing alleles to be dominant or recessive. The dominance of masculinizing and feminizing alleles is given by parameters $h_{m}$ and $h_{f}$ . We only consider completely dominant or completely recessive feminizing and masculinizing alleles (i.e. $h_{f}\in \left\{0,1\right\}$ and $h_{m}\in \left\{0,1\right\}$ ).

We take account of cosexuals that are capable of selfing by introducing a parameter of ‘fixed selfing’ where a fraction, θ , of eggs/ovules is fertilized by selfing and the remaining fraction is fertilized by outcrossing [43]. The offspring sired by selfing can suffer from inbreeding depression ( δ ), which reduces their survival probability. We assume that selfing does not reduce male competitive ability of cosexuals for outcrossing, which has been called ‘no pollen discounting’ [44–48]. Because we look at fully feminizing and masculinizing alleles, the male and female phenotypes cannot self. However, males and females may have increased male or female fitness, e.g. owing to the re-allocation of resources that are used for the other sexual functions in cosexuals. The relative increase in female/male sexual function in males/females relative to cosexuals is called ‘compensation’ and is given by the parameter $k_{f}$ for females and $k_{m}$ for males (equivalent to k and K in the notation of Charlesworth & Charlesworth [9]). For reference, the parameters are summarized in table 1.

**Table 1.** Parameter description.

parameter	description
$k_{f}$ / $k_{m}$	relative increase in male/female function for females/males (compensation)
R	recombination rate between loci
θ	selfing rate
δ	inbreeding depression
$h_{f}$ / $h_{m}$	dominance of feminizing/masculinizing mutation (assumed 0 or 1)
$e_{f}$ / $e_{m}$	determines whether the double mutant phenotype is female or male (assumed 0 or 1)

The crucial feature of our model is the double mutant phenotype, which is determined by $e_{f}\in \left\{0,1\right\}$ and $e_{m}\in \left\{0,1\right\}$ . These parameters allow us to consider the following three scenarios: the double mutant is sterile when $e_{f}=e_{m}=0$ , female when $e_{f}=1$ and $e_{m}=0$ and male when $e_{f}=0$ and $e_{m}=1$ . Note that we do not consider $e_{f}=e_{m}=1$ because this gives a cosexual double mutant that does not outcross (e.g. self=incompatibility). We only allow completely masculinizing and feminizing alleles, but smaller effect alleles are presumed to be subject to similar evolutionary forces with further assumptions required [11]. A fully general treatment could allow different selfing rates and male/female fitness for all nine genotypes (a total of 25 parameters) and is beyond the scope of this study. Table 2 summarizes the phenotypic effect of feminizing and masculinizing alleles given our model of dominance (parameters $h_{f}$ and $h_{m}$ ), selfing (parameter θ ) compensation (parameters $k_{f}$ and $k_{m}$ ) and double mutant phenotype (parameters $e_{f}$ and $e_{m}$ ).

**Table 2.** Selfing rates and fitness terms for different genotypes.

genotype	selfing	female fitness	female fitness
*ffmm*	θ	1	1
*Ffmm*	$\theta \left(1-h_{f}\right)$	$\left(1+h_{f}k_{f}\right)$	$\left(1-h_{f}\right)$
*FFmm*	0	$\left(1+k_{f}\right)$	0
*ffMm*	$\theta \left(1-h_{m}\right)$	$\left(1-h_{m}\right)$	$\left(1+h_{m}k_{m}\right)$
*FfMm*	$\theta \left(1-h_{f}\right)\left(1-h_{m}\right)$	$\left(1-h_{f}\right)\left(1-h_{m}\right)+$ $\left(1+k_{f}\right)h_{f}\left(1-h_{m}\right)+$ $\left(1+k_{f}\right)h_{f}h_{m}e_{f}$	$\left(1-h_{f}\right)\left(1-h_{m}\right)+$ $\left(1+k_{m}\right)h_{m}\left(1-h_{f}\right)+$ $\left(1+k_{m}\right)h_{f}h_{m}e_{m}$
*FFMm*	0	$\left(1+k_{f}\right)\left(1-h_{m}\right)+$ $\left(1+k_{f}\right)h_{m}e_{f}$	$\left(1+k_{m}\right)h_{m}e_{m}$
*ffMM*	0	0	$\left(1+k_{m}\right)$
*FfMM*	0	$\left(1+k_{f}\right)h_{f}e_{f}$	$\left(1+k_{m}\right)\left(1-h_{f}\right)+$ $\left(1+k_{m}\right)h_{f}e_{m}$
*FFMM*	0	$\left(1+k_{f}\right)e_{f}$	$\left(1+k_{m}\right)e_{m}$

To evaluate the spread of feminizing and masculinizing alleles, we track the frequency of the nine diploid genotypes. First, haploid gametes/gametophytes are produced with recombination between loci at rate R . When offspring are produced through selfing, the female and male gametes are derived from the same diploid individual. Owing to inbreeding depression, only a fraction (1-δ) of offspring produced by selfing survive to become reproductive adults in the next generation. Otherwise, fertilization occurs by sampling pairs of haploid genotypes to produce the diploid genotypes in the next generation. We provide a Mathematica [49] file that can be used to replicate our analyses (see electronic supplementary material). Where we were unable to obtain analytical results, we also used numerical iteration of the genotype frequency recursion equations. Our numerical iterations were implemented using R v.4.0.0 [50] and the scripts are provided in the electronic supplementary material.

## 3. Results

We first evaluate the spread of a rare feminizing or masculinizing allele in a population of cosexuals (with alleles *f* and *m* fixed). Specifically, we use the system of equations that describes the genotype frequency of all carriers of the *F* or *M* allele. The *F* or *M* allele spreads when the leading eigenvalue of this system is greater than one. As given by eqns (4) and (8) in Charlesworth & Charlesworth [9], a feminizer can invade an ancestrally cosexual population if


(3.1) $$1+k_{f}>2\left(1-\theta \;\delta \right),$$

whereas a masculinizer can invade if


(3.2) $$1+k_{m}>\frac{2\left(1-\theta \;\delta \right)}{1-\theta }.$$

**Figure 1.**
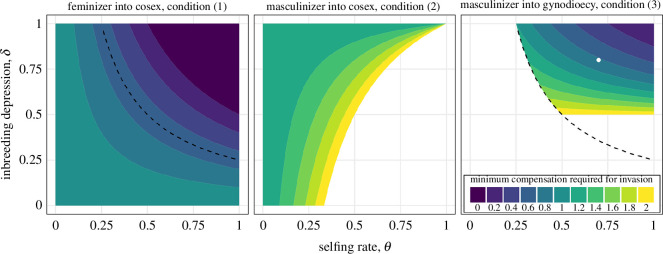
Conditions for invasion of a feminizing or masculinizing allele. Compensation is the increase in female/male sexual function in females/males relative to cosexuals. To show the invasion of masculinizers into gynodioecious populations, we assume that the compensation of the feminizer is $k_{f}=1/2$ , and the region where gynodioecy establishes is shown by a dashed line. The white dot shows the parameters used for figure 2.

These conditions apply to feminizers/masculinizers that are either dominant or recessive. Comparing equations (3.1) and (3.2) shows that females can invade a cosexual population with a smaller increase in sexual function than males (figure 1). This is because rare males in cosexual populations experience a lot of competition to fertilize the small number of eggs/ovules that are not fertilized by selfing.

**Figure 2.**
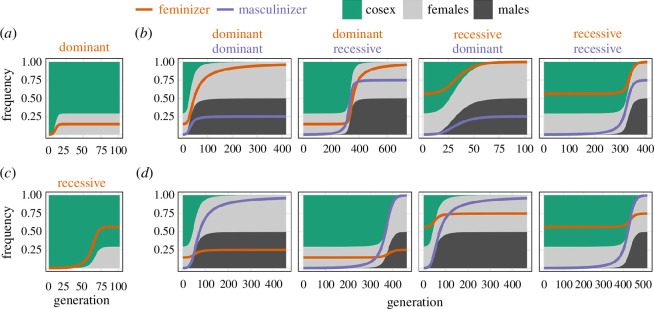
Examples of transitions from ancestral cosexuality to gynodioecious populations of females and cosexuals (*a,c*) and then to dioecious populations of females and males (*b,d*). The end state after the establishment of a dominant feminizer (*a*) or a recessive feminizer (*c*) is the starting point for subsequent invasion by a masculinizer in (*b,d*). The double mutant can be either male (*b*) or female (*d*), which have panels for different dominance combinations. Alleles with recessive effects are slower to establish when rare, but dominant alleles are slow to reach fixation because rare alleles are mostly found in heterozygotes. The parameters used are θ=7/10 , δ=8/10 , $k_{f}=1/2$ , $k_{m}=1$ and R=0.5 . The feminizing and masculinizing alleles were introduced at a frequency of 1/1000, and the simulations run for at least 100 generations and end when allele frequencies change by less than 0.0001.

We then evaluate the spread of a masculinizer in a gynodioecious population of females and cosexuals. We allow the double mutant to be either female ( $e_{f}=1$ and $e_{m}=0$ ) or male ( $e_{f}=0$ and $e_{m}=1$ ) and consider the four dominance combinations ( $h_{f}$ and $h_{m}$ ). We were only able to find analytical conditions for invasion by the masculinizer under three of these parameter combinations: (i) when the masculinizer is dominant and the feminizer is recessive and the double mutant is female ( $h_{m}=1$ , $h_{f}=0$ , $e_{f}=1$ and $e_{m}=0$ ), (ii) when both masculinizer and feminizer are dominant and the double mutant is female ( $h_{m}=h_{f}=e_{f}=1$ and $e_{m}=0$ ), and (iii) when both masculinizer and feminizer are dominant and the double mutant is male ( $h_{m}=h_{f}=e_{m}=1$ and $e_{f}=0$ ). Table 3 summarizes the dominance and double mutant phenotype combinations for which we have analytical results. All these cases have the same condition for the masculinizer to invade


(3.3) $$k_{m}>\frac{1+\left(1-2\delta \right)\theta }{k_{f}-\left(1-2\delta \right)\theta },$$

**Table 3.** Sex determination systems that evolve with different dominance and double mutant phenotypes. All analytical results are summarized using footnotes; other states are inferred from numerical iteration. Footnotes a, b and d correspond to results in Charlesworth & Charlesworth [9].

double mutant phenotype	feminizer^a^	masculinizer^b^	resulting sex determination system	male genotype X/Y or Z/Z	female genotype X/X or Z/W
sterile	dominant	dominant^c^			
	dominant	recessive	ZW (if R=0 )	*fM*/*fM*	*fM*/*Fm*
	recessive	dominant^d^	XY (if R=0 )	*Fm*/*fM*	*Fm*/*Fm*
	recessive	recessive			
male	dominant	dominant^e^	XY^f^	*Fm*/*FM*	*Fm*/*Fm*
	dominant	recessive	ZW^f^	*FM*/*FM*	*FM*/*Fm*
	recessive	dominant	XY^g^	*Fm*/*FM*	*Fm*/*Fm*
	recessive	recessive	ZW	*FM*/*FM*	*FM*/*Fm*
female	dominant	dominant^e^	ZW^f^	*fM*/*fM*	*fM*/*FM*
	dominant	recessive	ZW^g^	*fM*/*fM*	*fM*/*FM*
	recessive	dominant^e^	XY^f^	*FM*/*fM*	*FM*/*FM*
	recessive	recessive	XY	*FM*/*fM*	*FM*/*FM*

^a^ If feminizer is first mutation, then invasion is given by condition (1).

^b^ If masculinizer is first mutation, then invasion is given by condition (2).

^c^ Invasion into gynodioecy given by condition (A1) (see electronic supplementary material).

^d^ Invasion into gynodioecy given by condition (3) when R=0 .

^e^ Invasion into gynodioecy given by condition (3) with no restriction on recombination rate, R .

^f^ Stable given condition (1) is met.

^g^ Stable given conditions (1) and (3) are met.

which is equivalent to eqn (10) in Charlesworth & Charlesworth [9] (N.B., we assume complete loss of female function, i.e. $k^{*}=1$ in the notation of Charlesworth & Charlesworth [9]). The presence of females, whose eggs/ovules are available for fertilization through outcrossing, makes it easier for a masculinizer to invade such that condition (3) is more permissive than condition (2) (figure 1).

The case where the double mutant is sterile ( $e_{f}=0$ and $e_{m}=0$ ) is discussed in more detail in the electronic supplementary material. First, we find a new condition (A1) for a dominant masculinizer ( $h_{m}=1$ ) to invade a gynodioecious population with a dominant feminizer ( $h_{f}=1$ ) with double mutant sterility ( $e_{f}=0$ and $e_{m}=0$ ), which cannot result in dioecy. For a gynodioecious population with a recessive feminizer ( $h_{f}=0$ ), it has been shown [9] that condition (3) allows invasion by a dominant masculinizer ( $h_{m}=1$ ) that is completely linked ( R=0 , e.g. when both mutations occur in the same gene [12,13]) and appears on the non-feminizer haplotype. Increased recombination makes it more difficult for masculinizers to invade because it produces sterile double mutant genotypes. Thus, there can be a critical recombination rate above which masculinizers cannot invade, as calculated by eqn (11) in Charlesworth & Charlesworth [9]. These results are also summarized in table 3. An important distinction between the cases is that linkage is not a requirement when the double mutant is a fertile male or female, but it is required in cases where double mutants are sterile.

After the invasion of both the feminizing allele and the masculinizing allele, we expect the population to evolve towards dioecy with genetic sex determination. For example, when the double mutant is male, we expect the feminizing *F* allele to reach fixation with sex determined by variation at the other locus. If the masculinizing *M* allele is dominant, then it will act as a Y, with *Mm* males and *mm* females. To confirm that the system evolves towards the expected state, we conducted a new analysis where we evaluate the stability of dioecy to invasion by alleles that confer cosexuality (*f* and *m*). We found that the conditions required for the initial invasion by the feminizer and then masculinizer (conditions (1) and (3)) are typically sufficient for the stability of dioecy. We were able to show this in all cases except where both the masculinizer and the feminizer are recessive, as summarized in table 3.

We were not able to derive analytical invasion conditions for all dominance combinations (table 3), so we infer the dynamics of the other combinations using numerical iteration. This reveals a simple pattern. When the double mutant is male, the masculinizer remains polymorphic and determines sexual development, while the feminizer fixes (e.g. figure 2*c*). If the masculinizer is dominant, then it becomes a Y (reaching one-quarter frequency, with *Mm* males and *mm* females), whereas a recessive masculinizer becomes a Z (reaching three-quarters frequency, with *MM* males and *Mm* females). The reverse is true when the double mutant is female (figure 2*d*), in which case the masculinizer fixes and the dominance of the feminizer regulates the resulting sex determination system. A dominant feminizer becomes a W (reaching one-quarter frequency, with *ff* males and *Ff* females), whereas a recessive feminizer becomes an X (reaching three-quarters frequency, with *Ff* males and *FF* females). Figure 2 shows examples of these numerical simulations where the initially cosexual population evolves dioecy with different sex determination systems.

**Figure 3.**
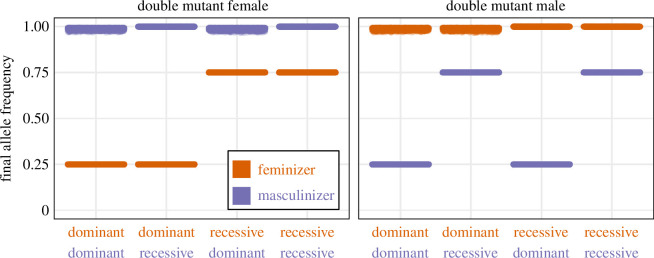
The dominance of the segregating locus dictates the sex determination system (XY or ZW). The final allele frequency of simulations is shown for different parameter combinations that favour transitions to dioecy (*n* = 5448 parameter combinations) for eight combinations of dominance and double mutant phenotype.

The numerical analysis in figure 2 only shows the sex determination systems that evolve for a particular set of parameters. To confirm that the inferred final sex determination systems are reached over a wider set of parameters, we chose 10 equally spaced values of θ , δ and $k_{f}$ between 0.1 and 1, 10 values of $k_{m}$ between 0.2 and 2 and two values of R (0.1 and 0.5), giving 20 000 possible parameter combinations. We were only interested in cases where invasion occurs, so we required that the invasion conditions were met, leaving 5448 parameter combinations for each of the eight combinations of dominance and double mutant phenotype. Specifically, we required that the two sides of condition (1) differed by at least 0.01 and that the sides of condition (3) differed by at least 0.1. These thresholds were chosen to avoid cases where the feminizing or masculinizing alleles are nearly neutral and spread very slowly, and the system does not reach equilibrium within a reasonable timeframe. We first introduced the feminizing allele and allowed the system to reach an equilibrium before introducing the masculinizing allele in linkage equilibrium with the feminizing allele. To speed up the simulations, the feminizing and masculinizing alleles were introduced at a frequency of 0.05 because alleles with recessive effects initially spread slowly (figure 2). All simulations ran for at least 100 generations and ended when the change in frequency of the masculinizing and feminizing alleles were less than $10^{-5}$ . We also required that the rate of change in allele frequency was declining. The frequencies of the masculinizing and feminizing alleles at the end of these numerical simulations show that, as expected, the masculinizer always goes to fixation when the double mutant is female, and *vice versa*, across all parameter combinations (figure 3). The resulting sex determination system then depends on the dominance of the segregating feminizer or masculinizer.

## 4. Discussion

Our models show that one-factor sex determination can evolve when the masculinizer and feminizer double mutant develops as either a male or a female. Specifically, we found that the conditions—in terms of selfing rate, inbreeding depression and male/female fitness gains—are the same for the establishment and stability of one-factor sex determination as they are for two-factor sex determination, except that the latter requires feminizing and masculinizing alleles to be linked on opposite haplotypes and have opposite dominance. On the other hand, the evolution of one-factor sex determination requires that the masculinizing and feminizing alleles interact to make the double mutant male or female.

One-factor sex determination systems, with sexual development controlled by variation at a single locus, are common. Most animals have one-factor sex determination, but terrestrial animals tend to have ancient dioecy, which makes it difficult to infer the early stages of sex chromosome evolution [4,22]. Transitions from cosexuality to dioecy in animals tend to occur via environmental sex determination [1], which may affect the evolution of new genetic sex determination systems. In flowering plants that have evolved dioecy from cosexuality, both one-factor and two-factor sex determination systems have evolved. Of 11 resolved sex determination systems (fig. 3*b* in Renner & Müller [16]), five species have two-factor systems and six have one-factor systems but this includes three *Populus* species and two *Salix* species with related sex determination systems, suggesting only two known origins of one-factor sex determination in flowering plants. In cases where the masculinizing and feminizing alleles have been uncovered, the different pathways to dioecy can be distinguished. In Caucasian persimmon, *D. lotus*, both the masculinizing and feminizing alleles are dominant and the double mutant is male [38,39]. Thus, the feminizing allele has reached fixation and the dominant masculinizing allele is now the sex-determining region on the Y chromosome [13,51], as predicted by our model.

In systems where the genetic details of sex determination are known, as in persimmon, sex determination systems can be categorized based on the dominance of masculinizing and feminizing alleles and the double mutant phenotype (table 3). This is most clearly demonstrated where dioecy has been created from ancestrally cosexual maize (*Zea mays*) [52,53] and melon (*Cucumis melo*) [54] by experimentally combining mutants. In maize, feminizing *tassel seed* alleles interact with the masculinizing *silkless* allele, making the double mutant female [52,53]. Dioecious maize populations have been created where the masculinizing allele is fixed and the recessive *tassel seed-2* feminizing allele segregates (creating XY sex determination) or the dominant *tassel seed-5* feminizing allele segregates (creating ZW sex determination) [55]. Similarly, dioecious populations of melon have been produced where the masculinizing allele is fixed because the double mutant is female; a segregating recessive feminizer then creates XY sex determination [54]. These experimental examples confirm how dominance and double mutant phenotype combine to create different sex-determining systems.

Either XY or ZW sex determination can evolve in our model and in previous models. However, most dioecious flowering plants have XY rather than ZW sex determination [18]. An explanation for this pattern has been advanced based on the classic model of two-factor sex determination [9]. The logic of this argument (see [5,10,18,56]) is that the feminizing allele is a sterility mutation and therefore most likely to be a recessive loss-of-function mutation. In the classic model—where the double mutant is sterile—this leads to XY two-factor sex determination after the spread of a masculinizing mutation (table 3 and figure A2 in the supplementary text). One difficulty with this explanation is that the masculinizing mutation must be dominant and occur in linkage to the feminizing allele, which are both presumed to be unlikely. Another issue is that only the relative likelihood of recessive/dominant mutations occurring is considered, ignoring their subsequent probability of establishment. The phenotypic effects of recessive alleles are hidden when rare, which means they segregate at low frequencies for a long time (figure 2) and can be lost through drift more easily. Thus, this is an incomplete explanation for the prevalence of XY sex determination. An alternative explanation has been advanced based on the evolving dominance of the sex-determining alleles [42]. The importance of dominance modification for the evolution of sex determination systems is also unknown, but this recent hypothesis may stimulate study in this area. In our model, the prevalence of XY versus ZW sex determination depends on the combination of dominance and double mutant phenotypes (table 3 and figures 2 and 3). For example, if feminizer and masculinizer double mutants tend to be female (e.g. [41]) and feminizing alleles tend to be recessive, then we expect XY sex determination to evolve more often.

Our model presents new evolutionary pathways whereby one-factor sex determination evolves during the early stages of sex chromosome evolution. The eventual sex determination system is regulated by the dominance and double mutant phenotype of feminizing and masculinizing alleles (table 3). These genetic details are likely to vary across species, and the available evidence suggests that different pathways have been followed in different species. Further empirical data may reveal circumstances that tend to be associated with specific evolutionary pathways, or the genetic details may be idiosyncratic. In any case, our results highlight that several alternative evolutionary routes can lead to genetic sex determination.

## Data Availability

An interactive Mathematica file to replicate our analyses is available as electronic supplementary material [57]. It also provides R scripts that can be used to replicate our numerical simulations and create the figures.
